# Understanding Pediatric Stroke: Etiology, Diagnosis, and Management in Children Under 15 Years

**DOI:** 10.7759/cureus.103412

**Published:** 2026-02-11

**Authors:** Dana AlZamer, Yazan S Al-Zamer, Majeed Al-Khafaji, Yousif M Basheer, Fatma Ahmed, Hameed Al-Khafaji, Basheer Basheer, Mohamed ElSerafi

**Affiliations:** 1 Family Medicine, Jordan University of Science and Technology, Irbid, JOR; 2 General Surgery, King Abdullah University Hospital, Irbid, JOR; 3 General Surgery, Jordan University of Science and Technology, Irbid, JOR; 4 Clinical Attachment, Al-Yarmouk Hospital, Irbid, JOR; 5 Medical School, Ajman University, Ajman, ARE; 6 Internal Medicine, Ajman University, Ajman, ARE; 7 Medical School, Jordan University of Science and Technology, Irbid, JOR; 8 Hospital-Based Medicine, HMS Al Garhoud Hospital, Dubai, ARE; 9 Neurology, HMS Al Garhoud Hospital, Dubai, ARE

**Keywords:** brain ischemia, cerebral hemorrhage, hypercoagulability, pediatric stroke, stroke management

## Abstract

Although rare in children under the age of 15, stroke is associated with significant mortality and long-term neurological morbidity. Understanding its underlying etiologies, accurate diagnostic modalities, and appropriate management is crucial for improving patient outcomes.

This narrative review article aims to summarize the current literature discussing the etiology, diagnosis, and management of stroke in children aged 15 years old or younger.

A comprehensive literature review was done using scientific databases such as PubMed, Scopus, and Google Scholar. The literature search included publications from 1997 to 2023, including peer-reviewed articles that specifically targeted pediatric stroke in patients under the age of 15 years. Only articles that specifically mentioned pediatric stroke were considered, excluding articles that specifically targeted adult patients, with a focus on more recent evidence in the management and therapeutic sections.

The review identifies cardioembolic sources, vascular abnormalities, hypercoagulable states, and infections as major etiologies of ischemic stroke in children. Hemorrhagic strokes, though less common, are primarily caused by trauma, aneurysms, and coagulopathies. While imaging modalities such as MRI and MR angiography have enhanced diagnostic accuracy, their availability is limited in resource-poor settings. Management is complicated by limited pediatric evidence supporting thrombolysis in pediatric ischemic stroke and the limited applicability of adult treatment guidelines.

Pediatric stroke is a rare but formidable diagnostic and therapeutic challenge due to its varied etiologies and clinical presentations. Early recognition and intervention are key to improving outcomes. Future research should focus on refining diagnostic strategies and developing targeted therapies for pediatric patients.

## Introduction and background

Introduction

Pediatric stroke is defined as cerebrovascular events in children younger than 15 years old. Although rare, its estimated annual incidence is 2-5 cases per 100,000 children; however, during the neonatal period, reported incidence is higher at 20-40 cases per 100,000 live births [1,2]. Despite the rarity of the condition, pediatric stroke represents an important cause of chronic neurological morbidity, with over half of the affected children going on to develop persistent neurodevelopmental impairments, including motor deficits, cognitive dysfunction, and epilepsy [1,3,4].

The etiologies of strokes in children differ significantly from those in adults. Atherosclerosis remains a leading cause in adults, whereas in children, the causes are varied and include congenital heart disease, non-arteriosclerotic arteriopathies, prothrombotic disorders, infectious diseases, and genetic anomalies [1,3,5]. In newborn infants, risk factors may also include placental diseases, infection around the time of birth, trauma, or hypoxic-ischemic encephalopathy [1,5]. Unlike in adults, the incidence of ischemic and hemorrhagic stroke in children varies with age; hemorrhagic stroke is more common in neonates and ischemic stroke in older children, which makes diagnosis and treatment more complicated [2,4].

Clinical presentations are age-related and can be nonspecific, especially in neonates and young children who cannot express their neurological symptoms. Presentations can include seizures, hemiparesis, changes in level of consciousness, and disturbances in speech or vision. A diagnostic challenge is the high prevalence of stroke mimics in children, including migraines, epilepsy, and metabolic disorders [2,3].

Making a timely diagnosis requires a high index of suspicion as well as neuroimaging studies. Among neuroimaging techniques, magnetic resonance imaging is considered the imaging modality of choice because of its high sensitivity for visualizing ischemic and hemorrhagic lesions and for differentiating stroke from stroke mimics [5]. Computed tomography can be employed in the initial assessment to rule out intracranial hemorrhage, although a negative scan does not rule out ischemic stroke in a child [5].

Management of pediatric stroke is limited by the absence of pediatric-specific evidence. This leads to reliance on adapted adult treatment protocols [1,5]. As a result, care centers on supportive measures, therapy targeting the underlying cause, prevention of further strokes, and early multidisciplinary rehabilitation to lessen long-term issues [1,5]. Despite the increasing awareness of pediatric stroke, gaps exist in pediatric-specific evidence, standardized diagnostic approaches, and age-specific treatment strategies. This manuscript reviews existing literature on pediatric stroke, examining its causes, diagnostic methods, and treatment approaches.

Methodology

This narrative review was conducted through a focused search of the existing literature using PubMed, Scopus, and Google Scholar databases. Relevant articles published in English were identified using keywords including pediatric stroke, childhood ischemic stroke, hemorrhagic stroke in children, stroke diagnosis, and stroke management. Original research articles, systematic reviews, clinical guidelines, and key review articles addressing stroke in children younger than 15 years were included. Studies focusing exclusively on adults and non-relevant case reports were excluded unless they provided mechanistic or clinical insights applicable to pediatric populations. The selected literature was reviewed and synthesized to provide an updated overview of the causes, diagnostic approaches, and treatment strategies for pediatric stroke. 

## Review

Pathophysiology and types of pediatric stroke

Stroke in children is stratified based on the underlying pathophysiology into two separate entities, one of which is ischemic stroke, where the brain perfusing arterial blood flow supply is blocked, and brain parenchymal tissue distal to the arterial occlusion is deprived of adequate blood supply [4]. The other stroke entity is hemorrhagic stroke, which is caused by the disruption of the blood vessels carrying blood towards or away from the brain. This disruption allows blood to extravasate into the brain parenchyma or into the surrounding meningeal spaces [5]. Table 1 summarizes the main categories of pediatric stroke and their associated etiologies.

**Table 1:** Classification and Major Etiologies of Pediatric Stroke

Type	Subtype	Major Etiologies
Arterial	Arterial ischemic stroke	Congenital heart disease, arteriopathies, prothrombotic states, infection
Venous	Cerebral venous thrombosis	Prothrombotic disorders, dehydration, infection
Hemorrhagic	Intracerebral hemorrhage	Vascular malformations, trauma, coagulopathies
	Subarachnoid hemorrhage	Aneurysms, vascular malformations

Underlying etiologies for pediatric ischemic stroke

Ischemic stroke represents the predominant subtype of stroke in the pediatric population [5]. Other etiologies are discussed in the following sections. 

Cardioembolic Sources

Cardioembolic causes of stroke in children include cardiac arrhythmias, rheumatic heart disease, valve disease, prosthetic valves, and patent foramen ovale (PFO). Cardiac arrhythmias, such as atrial fibrillation, can lead to clot formation, which may embolize to the cerebral circulation [6]. Rheumatic heart disease can cause valvular abnormalities, predisposing children to embolic events. Valve diseases, such as subacute infective endocarditis, can damage heart valves, leading to clot formation and subsequent stroke. Prosthetic valves increase the risk of thromboembolism due to their thrombogenic nature. Finally, PFO, though less common, can serve as a conduit for paradoxical emboli, allowing venous clots to bypass the pulmonary circulation and enter the systemic circulation, potentially causing stroke [7].

Hypercoagulable Status

Hereditary and acquired hypercoagulable conditions predispose children to thrombosis and ischemic stroke. These include factor V Leiden mutation, a common hereditary thrombophilia that increases the risk of venous and arterial thrombosis; Protein C/S, Antithrombin III deficiencies, which reduce natural anticoagulant activity; antiphospholipid syndrome, an autoimmune disorder associated with recurrent thrombosis; and sickle cell disease, a hemoglobinopathy that increases blood viscosity and predisposes to vascular occlusion [8].

Vascular Disorders

Vascular malformations, particularly pulmonary arteriovenous malformations (AVMs), can increase the risk of ischemic stroke. Pulmonary AVMs act as a conduit for venous thrombi to enter the systemic circulation, leading to embolic strokes. Other vascular causes include cervical arterial dissection, which can result from spontaneous or trauma-induced tears in the cervical vessel walls, and Moyamoya disease, a rare condition characterized by progressive stenosis of intracranial arteries, predisposing children to ischemic stroke [9].

Infections

Infections affecting the central nervous system, such as meningitis, including both viral and bacterial entities alongside other infections affecting the nervous system, could lead to vasculitis, predisposing these children to the development of arterial thrombosis followed by ischemic stroke [10,11].

Iron Deficiency Anemia

This condition is a lesser-known but significant risk factor for ischemic stroke in children, particularly in severe cases. Iron deficiency anemia promotes a hypercoagulable state by increasing platelet aggregation and blood viscosity. Additionally, impaired oxygen delivery exacerbates cerebral ischemia [12].

Underlying etiologies for pediatric hemorrhagic stroke

Formed as a result of the disruption of cerebral vessels and intraparenchymal bleeding, hemorrhagic strokes represent a less common category of pediatric strokes in terms of incidence [13]. A summary of the causes of hemorrhagic strokes is described in the following sections. 

Intracranial Vascular Aneurysms, Arteriovenous Malformations, and Arteriovenous Fistulas

Congenital vascular malformations and aneurysms within cerebral vessels are naturally more susceptible to spontaneous rupture due to the weak integrity of vascular walls, which can lead to the development of hemorrhagic stroke. These vascular malformations could be a result of inborn congenital disorders within the constituents that form vessel walls, such as collagen disorders, or acquired throughout the child's life [14].

Trauma

Exposure of children to blunt force or external trauma may lead to the development of hemorrhagic stroke, mainly due to a lower amount of brain parenchyma and a larger amount of fluid surrounding the brain when compared to adults. This leads to a more movable and agile vessel that could stretch out and tear, increasing the risk of hemorrhagic stroke [15].

Coagulopathies

Inherited or acquired bleeding disorders predispose children to hemorrhagic stroke. Examples include hemophilia, congenital vitamin K deficiency, von Willebrand disease, and congenital thrombocytopenia [16-18].

Diagnostic challenges and approaches

Pediatric strokes typically present with subtler or atypical patterns compared to adult stroke presentations, creating diagnostic challenges for physicians. Moreover, children are less likely to communicate their symptoms effectively, further complicating the diagnosis process [19].

Clinical Presentation for Pediatric Stroke

Older children who experience strokes may present with symptoms similar to those of a stroke in adults, causing focal neurological deficits, including hemiparesis, speech impairment, limb weakness, facial weakness, and others, depending on the exact location affected by the stroke incident. On the other hand, younger children and infants' strokes may present with less prominent neurological manifestations such as seizures, regression of neurological development, and falling behind chronologically expected milestones, or presenting as an altered level of consciousness [18-20].

Imaging Modalities Utilized

Physicians suspecting stroke in children usually resort to using a computed tomography (CT) scan of the brain initially to exclude the hemorrhagic type of stroke, given its high sensitivity for hemorrhage, and the urgency to rule out said type. CT scans are less sensitive for ischemic strokes; thus, physicians resort to the utilization of magnetic resonance imaging (MRI) with diffusion-weighted imaging to diagnose an ischemic stroke, given that this is the gold standard due to the high sensitivity for ischemic stroke and stroke mimics, and may be required before reperfusion therapies. MR angiography of the head and neck is also performed with brain MRI to evaluate vascular lesions, including thrombus, emboli, and dissections, and when the physician has a suspicion for any vascular abnormalities, to identify the source of cerebral ischemia [21,22].

Laboratory Investigations

Pediatric stroke patients usually should undergo a comprehensive panel of routine blood tests to evaluate potential underlying causes. These include diseases that predispose to a hypercoagulable state, such as factor V Leiden mutation, lupus anticoagulant, protein C and S deficiency, and other thromophilias. An echocardiogram is also used to rule out a cardiac source for an embolus, especially in children who have been diagnosed with a congenital heart defect or suspected to have a patent foramen ovale. Genetic testing for heritable disorders is also used to investigate inherited disorders such as Moyamoya disease, cerebral autosomal dominant arteriopathy with subcortical infarcts and leukoencephalopathy (CADASIL), and Fabry disease, which may present with stroke at a young age. Comprehensive metabolic panels can also help identify metabolic disorders contributing to stroke [6-8].

Barriers in Resource-Limited Settings

In resource-poor settings, the inaccessibility of advanced imaging modalities such as MRI and MR angiography contributes to delayed diagnosis and poor outcomes. Similarly, in most instances, laboratory facilities for the investigation of rare coagulation disorders or metabolic etiologies may not be available for comprehensive evaluation [22].

Management of pediatric stroke

Pediatric stroke management requires a multidisciplinary team approach with the pertinent collaborative work shared among neurologists, intensivists, rehabilitation specialists, and psychologists. Treatment strategies depend on the type of stroke (ischemic or hemorrhagic) and the underlying etiology causing or provoking stroke development [5].

Acute Management of Ischemic Stroke

Children with acute ischemic stroke are typically managed in an intensive care unit, where supportive care is provided. This includes treatment of hyperglycemia, identification and treatment of hyperthermia, and management of hypotension [23].

Intravenous thrombolytic therapy with recombinant tissue plasminogen activator is used less frequently in children than in adults, due to limited safety and efficacy data [23]. Mechanical thrombectomy has emerged as a potential therapeutic option for selected children with large-vessel occlusion; however, its use remains limited and may be considered in highly selected pediatric patients, although current evidence is largely derived from observational studies [24]. In addition, direct oral anticoagulants are being explored in selected cases of pediatric stroke with confirmed cardioembolic etiology [7].

Acute Management of Hemorrhagic Stroke

Management of hemorrhagic stroke in children is directed toward controlling intracranial pressure and preventing secondary brain injury. This includes medical therapies such as osmotic agents (e.g., mannitol) and, when appropriate, controlled hyperventilation to reduce the risk of cerebral herniation. When a vascular malformation or aneurysm is identified as the underlying cause, surgical intervention may be required [23].

Rehabilitation and Long-Term Care

**Figure 1:**
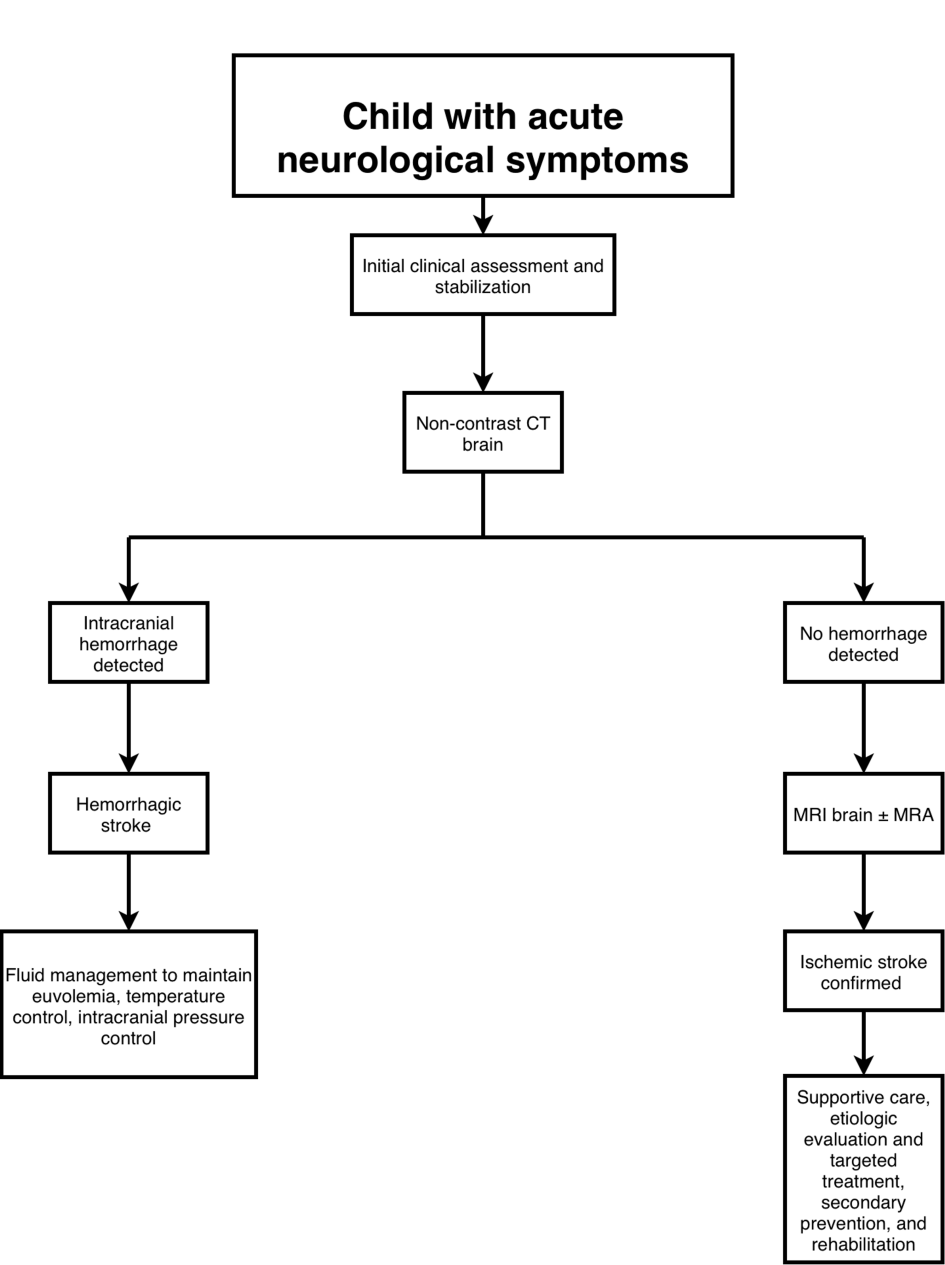
Diagnostic and Management Approach to Suspected Pediatric Stroke Simplified diagnostic and management pathway for children presenting with acute neurological symptoms suggestive of stroke, highlighting early neuroimaging, differentiation between hemorrhagic and ischemic stroke, and etiology-directed management with secondary prevention and rehabilitation. Figure created by the authors from the reviewed literature, using draw.io

Rehabilitation is the cornerstone of improving outcomes for pediatric stroke survivors. Early initiation of physical therapy, speech therapy, and occupational therapy reduces long-term disability and promotes functional independence. Long-term follow-up, including age-appropriate neuropsychological evaluations to define educational and support needs, must be done regularly, and monitoring for recurrent stroke complications is necessary [25]. Figure 1 illustrates a simplified diagnostic and management approach to suspected pediatric stroke.

## Conclusions

Pediatric stroke presents diagnostic and management challenges due to distinct etiologies, clinical presentations, and long-term neurological outcomes compared with adult stroke. The findings from this literature review highlight the importance of early recognition and timely intervention in order to improve outcomes in affected children. Stroke in children is a complex condition with diverse causes, ranging from cardio-embolic sources, vascular disorders, and hypercoagulable states, necessitating a comprehensive diagnostic and therapeutic approach. The literature points out the existing gaps in pediatric stroke management, such as the delay in diagnosis, the lack of evidence for acute management, and the lack of guidelines for pediatric patients. To fill these gaps, there will be a need for collaboration in research to establish guidelines for pediatric patients, while the index of suspicion for stroke should remain high in pediatric patients presenting with acute neurological symptoms.
